# The identification of tumor antigens and immune subtypes based on the development of immunotherapies targeting head and neck squamous cell carcinomas resulting from periodontal disease

**DOI:** 10.3389/fonc.2023.1256105

**Published:** 2023-08-22

**Authors:** Yangju Fu, Yongbo Zheng

**Affiliations:** ^1^ Operating Room, West China Hospital, Sichuan University/West China School of Nursing, Sichuan University, Chengdu, China; ^2^ Department of Otolaryngology Head and Neck Surgery, West China Hospital, Sichuan University, Chengdu, China

**Keywords:** head and neck squamous cell carcinoma, periodontal disease, immune subtypes, ADM, tumor antigen

## Abstract

Among cancer treatments, immunotherapy is considered a promising strategy. Nonetheless, only a small number of individuals with head and neck squamous cell carcinoma exhibit positive responses to immunotherapy. This study aims to discover possible antigens for head and neck squamous cell carcinoma, create an mRNA vaccine for this type of cancer, investigate the connection between head and neck squamous cell carcinoma and periodontal disease, and determine the immune subtype of cells affected by head and neck squamous cell carcinoma. To ascertain gene expression profiles and clinical data corresponding to them, an examination was carried out on the TCGA database. Antigen-presenting cells were detected using TIMER. Targeting six immune-related genes (CXCL5, ADM, FGF9, AIMP1, STC1, and CDKN2A) in individuals diagnosed with head and neck squamous cell carcinoma has shown promising results in immunotherapy triggered by periodontal disease. These genes have been linked to improved prognosis and increased immune cell infiltration. Additionally, CXCL5, ADM, FGF9, AIMP1, STC1, and CDKN2A exhibited potential as antigens in the creation of an mRNA vaccine. A nomogram model containing ADM expression and tumor stage was constructed for clinical practice. To summarize, ADM shows potential as a candidate biomarker for predicting the prognosis, molecular features, and immune characteristics of head and neck squamous cell carcinoma cells. Our results, obtained through real-time PCR analysis, showed a significant upregulation of ADM in the SCC-25 cell line compared to the NOK-SI cell line. This suggests that ADM might be implicated in the pathogenesis of HNSC, highlighting the potential of ADM as a target in HNSC treatment. However, further research is needed to elucidate the functional role of ADM in HNSC. Our findings provide a basis for the further exploration of the molecular mechanisms underlying HNSC and could help develop novel therapeutic strategies.

## Introduction

In the mouth exist over 700 microorganisms, including symbiotic and opportunistic bacteria, viruses, and fungi that are in symbiotic relationships with each other (1). Infection with periodontal pathogens causes chronic inflammation of the periodontal tissues, resulting in damage to the supporting tissues of the teeth and ultimately tooth loss (2). Severe periodontitis affects approximately 10.8% of the overall populace, ranking as the sixth most common ailment globally (3). Multiple research studies have indicated that periodontal disease raises the likelihood of various malignancies, such as oral cancer, breast cancer, head and neck squamous cell carcinoma (HNSC), and prostate cancer (4). Furthermore, research has revealed that periodontal disease can impact overall health, such as atherosclerotic cardiovascular disease, diabetes, negative pregnancy outcomes, and chronic obstructive pulmonary disorders (5, 6). Significant impact on public health could occur if interventions that decrease the likelihood of developing or exacerbating periodontal disease also decrease the likelihood of developing or exacerbating cancer (7). As a result of the host immune-inflammatory response, environmental factors, genomics, and microenvironments, periodontal disease results in an imbalance of epithelial and connective tissue homeostasis (8). Interactions between these molecules result in enormous complexity, with dysregulation of the transcriptome, methylome, proteome, and metabolome, all of which may contribute to periodontal inflammation and tissue destruction (9).

In 2012, there were approximately 300,000 newly reported instances of HNSC globally, leading to a mortality rate of 2.7 per 100,000 individuals (10). The incidence has also increased among young and middle-aged individuals in recent years (11). According to reports, HNSC ranks as the sixth most prevalent cancer globally, with squamous cell carcinoma being the predominant type (12). Although there is a 50% probability of early detection of HNSC, the majority of individuals with HNSC receive a diagnosis during a late stage. Approximately 16% of individuals diagnosed with HNSC manage to survive for a complete period of five years as a result of either local or distant spread (13). It is disappointing that the clinical outcomes of patients have not improved significantly over the past decade despite advances in surgery, chemotherapy, and radiation therapy (14). HNSC is considered to have a multifactorial nature, with tobacco, alcohol, and betel nuts identified as the primary risk factors (15, 16).

There are two main types of immunotherapy for cancer: passive and active forms. Active immunotherapy involves stimulating the patient’s immune system, which results in the activation of natural killer cells, T cells, or the production of antibodies targeting tumor-specific antigens (17). In the meantime, the innate immune system is strengthened through passive immunotherapy, which involves temporarily introducing external proinflammatory cytokines into the body to revive the T helper cell (Th)-1 response (18). By activating immune checkpoint pathways, cancer cells are able to escape detection by the immune system. Blocking immune checkpoints can hinder the activation of the PD-1 immune checkpoint protein, enabling T cells to attack cancer cells and restore health. There is increasing evidence that these drugs inhibit the progression of some solid tumors in recent years (19). Nivolumab and pembrolizumab, among other newly developed drugs, work by inhibiting the immune checkpoint protein PD-1, which leads to the restoration of T cell function and their ability to combat cancer cells (20). Additionally, ongoing research is being conducted on PD-L1 inhibitors for the management of different types of tumors, including certain malignancies that are currently being treated using these inhibitors (21). Furthermore, the utilization of PD-1 and PD-L1 inhibitors in immunotherapy is linked to heart failure, along with other adverse effects like dermatitis, autoimmune enteropathy, thyroid abnormalities, and autoimmune liver inflammation (22). There is an urgent need for the advancement of more efficient immunotherapies for HNSC.

To comprehend the correlation between HNSC and periodontitis, a bioinformatics analysis was carried out as a component of this study. The TCGA database was utilized to retrieve mRNA expression profiles and clinical data of patients diagnosed with HNSC. Furthermore, the CTD repository was utilized to retrieve genes linked to periodontal disease. Through the analysis of GO and KEGG enrichment, it was found that periodontal disease is linked to HNSC. According to bioinformatic analysis, there is an indication of a strong association between periodontal disease and multiple other illnesses. Furthermore, the analysis of differential expression was conducted to investigate the correlation between periodontal disease and HNSC. Next, we developed prognostic indicators to further investigate the relationship between the genes associated with periodontal disease and HNSC. In order to validate the reliability of the prognostic prediction model, survival analysis and ROC curves were performed. As part of our research, we have successfully analyzed bioinformatics data on periodontal disease and HNSC. The present study presents a novel approach to investigating the correlation between periodontal disease and HNSC.

## Methods

### Dataset downloaded

The Comparative Toxicogenomics Database (CTD) (23–26) is a valuable resource for enhancing our knowledge of the impact of environmental exposures on human health. An information source that offers details on the connections among molecules, genes, and proteins, along with the associations between chemicals, diseases, and genes. To identify genes closely associated with the disease, we obtained the genes related to periodontal disease from the CTD database. The Tumor Genome Atlas (TCGA) was launched in 2006 through a collaboration between the National Cancer Institute (NCI) and the National Human Genome Research Institute (NHGRI), with funding provided by the National Cancer Institute. Extensive collaboration was employed to enhance comprehension of the molecular foundation of cancer by utilizing advanced genome analysis technology on a large scale. To further examine the genes associated with this type of cancer, we obtained transcript data and clinical information pertaining to HNSC. This study included a cohort of 504 individuals diagnosed with HNSC and 44 healthy controls. Moreover, the ImmPort repository held a grand total of 3178 genes associated with the immune system.

### The variation in expression of genes associated with periodontal disease and the immune system

In the first step, TCGA expression data for HNSC were downloaded. Using standardization, normalization, analysis, and processing, gene expression data were divided into groups representing HNSC and groups representing normal tissue. Furthermore, we acquired genes that have a strong correlation with periodontal disease and the immune system. Additionally, we classified genes as differentially expressed if their |log_2_FC| was greater than 1 and their p-value was less than 0.05.

### Perform enrichment analysis using GO and KEGG

Using the ClusterProfiler package, we annotated key genes and explored their functions. The relationships between the genes were explored using Gene Ontology (GO) and the Kyoto Encyclopedia of Genes and Genomes (KEGG). Statistical significance was determined when the p values of GO and KEGG enrichment pathways were both less than 0.05.

### Network of interactions between proteins (PPI)

The PPI network of interactive genes was generated using STRING (https://www.string-db.org/). Composite Ratings with values above 0.4 were deemed to be statistically significant. Furthermore, the PPI networks were examined and displayed using Cytoscape version 3.7.2.

### Construction of a gene-based prognostic index (IRPDGPI) for immune-related periodontal disease using a prognostic prediction model

In order to build a model that predicts prognosis, we utilized univariate and multivariate cox regressions, along with lasso regressions. For every patient, a risk score formula was developed by considering values from individual genes and assigning weights according to their estimated regression coefficients in the lasso regression analysis. A classification was made by using the risk score formula to divide the patients into two groups: one with low risk and another with high risk. The baseline was established using the median risk score value. The log-rank statistical approach was employed to compare the survival rates of the two groups using the Kaplan-Meier survival analysis. To assess the impact of risk score on the patient’s prognosis, a study employed Lasso regression and stratified analysis. ROC curves were utilized to assess the precision of model predictions. Additionally, the analysis of univariate and multivariate independent cox regression was employed to ascertain if IRPDGPI served as an autonomous prognostic factor in HNSC.

### Immune cell infiltration analysis

CIBERSORT, a widely utilized technique for assessing the cellular makeup through gene expression profiling, is frequently employed in the estimation and examination of immune cell infiltration. The CIBERSORT algorithm was utilized to analyze the RNA-seq data for determining the ratio of immune-infiltrating cells in both tumor and normal groups. Each sample had an identical score of 1 for all estimated immune cell types. Statistical significance was determined by performing a spearman correlation analysis between gene expression and immune responses in cell content, with a significance level of P<0.05.

### Examining the clinical significance and correlation analysis of the IRPDGPI

Clinical data including age, sex, grade, T, N, and M stages were obtained from the TCGA repository. Additionally, the DCA curve and nomogram were constructed using R studio. The evaluation of the correlation between IRPDGPI and clinical value was conducted using the ‘RColorBrewer’ package in R.

### Gene Set Enrichment Analysis (GSEA)

Gene sets were obtained using MSigDB, available at http://www.gsea-msigdb.org/gsea/downloads.jsp. Enriched GO terms and KEGG pathways were identified by conducting GSEA on the gene sets. We chose the most significant 50 terms from each subtype.

### Cell culture

The SCC-25 cell line, originating from human oral squamous carcinoma, was successfully re-established in our laboratory. These cells were sustained in a 1:1 blend of Dulbecco’s minimum essential medium and Ham’s F-12, enriched with 10% fetal bovine serum along with antibiotics, specifically 100 units/mL of penicillin and 100 μg/mL of streptomycin. The culturing conditions involved a humid environment with a controlled atmosphere of 5% CO2, held at a steady temperature of 37°C. Our laboratory successfully revitalized the NOK-SI cell line, comprising spontaneously immortalized human oral keratinocytes, sourced from normal tissue. These cells were nurtured in Dulbecco’s modified Eagle’s medium, enhanced with 10% fetal bovine serum (FBS) and 1% antibiotic/antimycotic combination. The culturing process was conducted at a constant temperature of 37°C within an atmosphere containing 5% CO2.

### Quantitative real-time PCR

Total RNA was extracted from cells using the RNeasy Mini Kit following the manufacturer’s instructions. The concentration and purity of the RNA samples were determined by NanoDrop spectrophotometer. Subsequently, complementary DNA (cDNA) was synthesized from 1 µg of total RNA using the High Capacity cDNA Reverse Transcription Kit, according to the manufacturer’s guidelines. Real-time PCR analysis was performed on a QuantStudio 5 Real-Time PCR System using PowerUp SYBR Green Master Mix. Each 20 µl reaction mixture contained 10 µl of SYBR Green Master Mix, 1 µl of forward primer, 1 µl of reverse primer, 1 µl of cDNA, and 7 µl of nuclease-free water. The thermal cycling conditions were set as follows: initial denaturation at 95°C for 10 minutes, followed by 40 cycles of 95°C for 15 seconds and 60°C for 1 minute. Relative gene expression was calculated using the 2^−ΔΔCt method, with GAPDH used as the endogenous reference gene.

### Statistical analysis

Survival curves were created and compared using the Kaplan-Meier technique, with log rank as the basis for comparison. The Cox proportional hazard model was utilized to perform the multivariate analysis. R (version 3.6) was utilized for conducting all statistical analyses. Obtaining a p-value of 0.05 in all statistical tests was deemed to be statistically significant.

## Results

### Differential expression analysis

**Figure 1:**
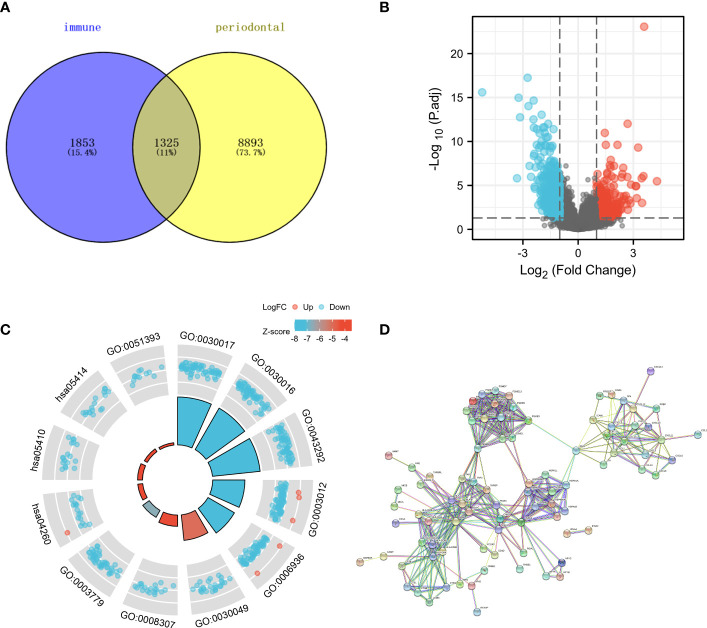
**(A)** The Venn diagram demonstrated the genes that are associated with immune and periodontal disease; **(B)** The volcano map showed the differential expression of HNSC; **(C)** The GO and KEGG enrichment analysis about the genes that are associated with immune and periodontal disease; **(D)** The PPI network of differential expression genes.

The online tool database reported that periodontal disease had close associations with 3178 genes, whereas immunity had close associations with 10218 genes. **Figure 1A** displayed a Venn diagram indicating that 1325 genes had a strong correlation with both periodontal disease and immune function. The analysis included a total of 548 samples, with 44 being normal samples and 504 being tumor samples obtained from patients diagnosed with HNSC (as shown in **Figure 1B**). There were 54492 genes whose expression level has been detected. Based on the findings, a total of 382 genes exhibited up-regulation in HNSC samples. In comparison to the normal sample, there was a decrease in the expression of 471 genes in the HNSC sample.

### Enhancing the genetic composition of genes associated with the immune system and genes implicated in periodontal disease

Various pathways showed significant enrichment of genes associated with immune response and periodontal disease, as indicated by the GO and KEGG enrichment analysis. For instance, the GO enrichment analysis indicated that several genes were linked to processes related to the muscular system, such as muscle contraction, sliding of muscle filaments, sarcomere, myofibril, contractile fiber, structural component of muscle, binding to actin, and binding to alpha-actinin (**Figure 1C**). Moreover, the KEGG enrichment analysis revealed the enrichment of numerous genes in pathways related to the contraction of cardiac muscle, hypertrophic cardiomyopathy, and dilated cardiomyopathy. Our research utilized the CTD and immune-related databases to detect interconnected genes associated with periodontal disease and immune response, forming a network of protein-protein interactions (PPI) among these genes. The findings indicated that several genes exhibited over 30 interactive counts, such as B2M, HSPA5, PSMB8, HLA-B, HLA-C, HLA-A, CANX, CALR, HLA-DRA, and CXCL10 (**Figure 1D**).

### Construction of IRPDGPI (prognostic index based on genes associated with immune-related periodontal disease)

**Figure 2:**
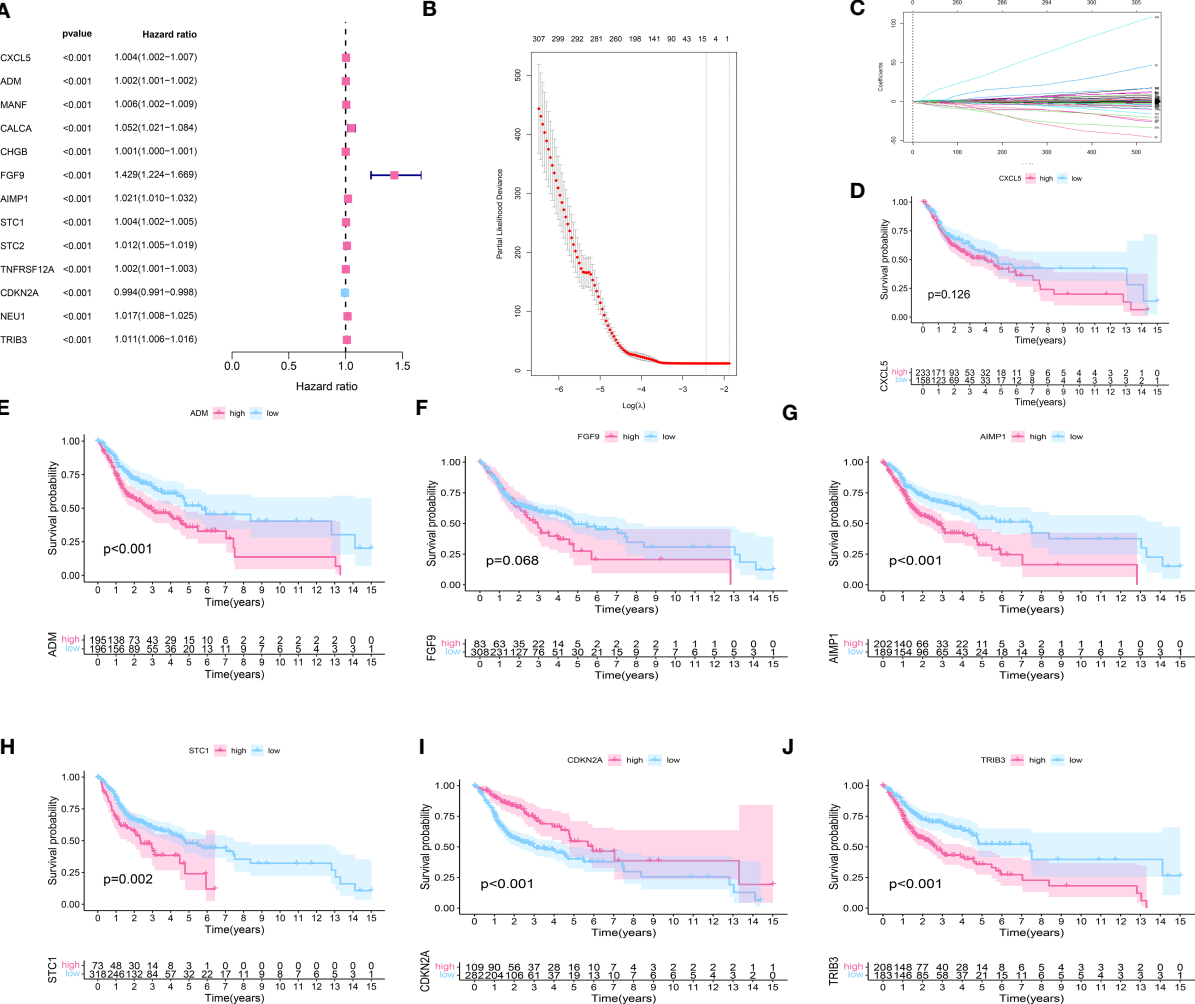
**(A)** The univariate cox regression about the HNSC data; **(B, C)** The lasso regression about the HNSC data; **(D)** The survival analysis of CXCL5; **(E)** The survival analysis of ADM; **(F)** The survival analysis of FGF9; **(G)** The survival analysis of AIMP1; **(H)** The survival analysis of STC1; **(I)** The survival analysis of CDKN2A; **(J)** The survival analysis of TRIB3.

**Figure 3:**
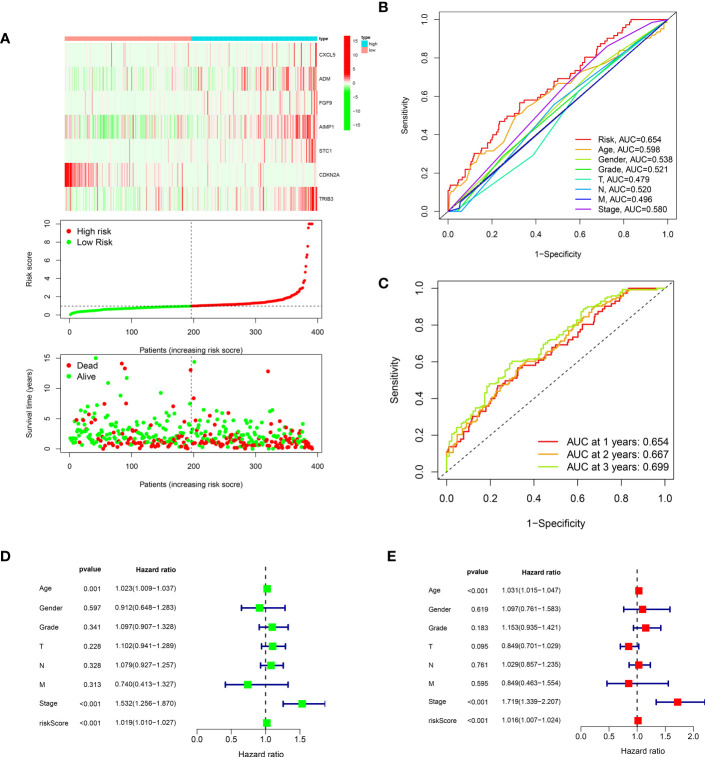
**(A)** The risk curve of IRPDGPI; **(B)** The clinic-related ROC curve; **(C)** The time-dependent ROC curve; **(D)** The univariate independent cox regression; **(E)** The multivariate independent cox regression.

To further investigate the genes associated with periodontal disease and HNSC, the clinical data of patients with HNSC was acquired. Next, we performed a univariate Cox regression analysis and a lasso regression analysis to discover the distinctive genes in HNSC. Based on the data, univariate Cox regression (**Figure 2A**) identified 13 genes associated with prognosis (p value<0.01). In the subsequent stage, we executed the multivariate cox regression analysis (**Figures 2B, C**). Following the multivariate cox regression, we acquired the optimal risk score (Risk Score = CXCL5 * 0.00341351711657313 + ADM * 0.000848728116009434 + FGF9 * 0.339265398865143 + AIMP1 * 0.0168592756657467 + STC1 * 0.00269346117976538 + CDKN2A * -0.00662950138257665 + TRIB3 * 0.00830904795288386) values for further examination. Based on the median risk score, patients were categorized into groups with low and high risk (**Figure 3A**). Furthermore, the prognosis of HNSC patients (P<0.001) (**Figures 2D–J**) was found to be strongly associated with ADM, AIMP1, STC1, CDKN2A, and TRIB3 according to the Kaplan-Meier curves. Furthermore, the results from the ROC curve demonstrate that the AUC for 1, 2, and 3 years surpasses 0.6, signifying a commendable rate of success in validating the model (**Figure 3C**). In order to evaluate the independent prognostic factors in HNSC, we conducted both univariate and multivariate independent prognostic analyses. Through the utilization of both univariate and multivariate independent prognosis analysis, it was discovered that age, stage, and risk score played significant roles as independent determinants in HNSC (as depicted in **Figures 3D, E**).

### IRPDGPI gene expression evaluation

**Figure 4:**
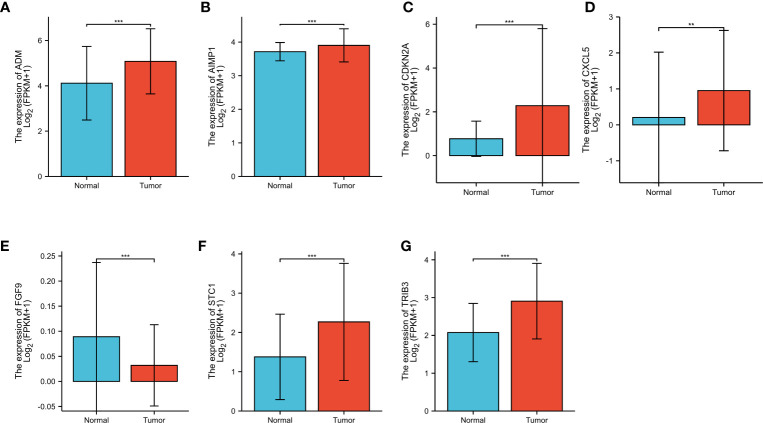
**(A)** The different expression analysis of ADM; **(B)** The different expression analysis of AIMP1; **(C)** The different expression analysis of CDKN2A; **(D)** The different expression analysis of CXCL5; **(E)** The different expression analysis of FGF9; **(F)** The different expression analysis of STC1; **(G)** The different expression analysis of TRIB3. ** represents for p < 0.01, *** represents for p < 0.001.

To investigate the gene expression level related to IRPDGPI, we conducted an analysis of ADM, CXCL5, ADM, FGF9, AIMP1, STC1, and CDKN2A expression in HNSC samples compared to normal samples. The findings indicated that there was a significant difference in the analysis of all 7 genes between the HNSC group and the normal group. ADM, AIMP1, CDKN2A, CXCL5, STC1, and TRIB3 exhibited increased expression in the HNSC group when compared to the normal group. In the HNSC group, the expression of FGF9 was decreased compared to the normal group as shown in **Figures 4A–G**.

### Identification of IRPDGPI genes at the immune cell level

**Figure 5:**
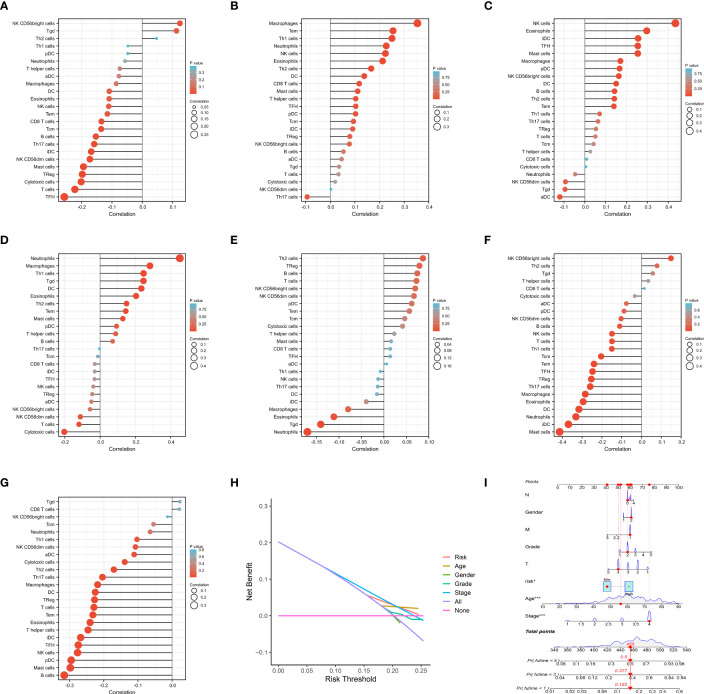
**(A)** The immune infiltration analysis of ADM; **(B)** The immune infiltration analysis of AIMP1; **(C)** The immune infiltration analysis of CDKN2A; **(D)** The immune infiltration analysis of CXCL5; **(E)** The immune infiltration analysis of FGF9; **(F)** The immune infiltration analysis of STC1; **(G)** The immune infiltration analysis of TRIB3. **(H)** The DCA curve of IRPDGPI and clinical information; **(I)** The nomogram of IRPDGPI and clinical information.

To classify the expression data, we considered the low and high levels of gene expression associated with IRPDGPI.ADM showed a positive correlation with NK CD56 bright cells and Th2 cells, while exhibiting a negative correlation with B cells, mast cells, pDC, TFH, iDC, T cells, and NK cells. AIMP1 showed a positive correlation with NK CD56 bright cells and T cells. Moreover, there was a favorable association observed between CDKN2A and B cells, T cells, CD56 bright cells, NK CD56 dim cells, as well as pDCs. Besides neutrophils, macrophages, Tgd, DS, eosinophils, T2 cells, mast cells, and pDCs, CXCL5 exhibited a favorable association with neutrophil activity. Furthermore, it has been demonstrated that FGF9 exhibits a positive correlation with Th2, Treg, B, T, NK, NKCD56 dim, and NKCD56 bright cells. In addition, we found that STC1 has a strong association with NK CD56 bright cells, Th2 cells, Tgd and T helper cells, whereas TRIB3 is closely linked to Tgd, CD8 T cells, NK CD56 bright cells, Tcm, neutrophils, and TGCs (as shown in **Figures 5A–G**).

### Clinical value of IRPDGPI studied by correlation analysis

**Figure 6:**
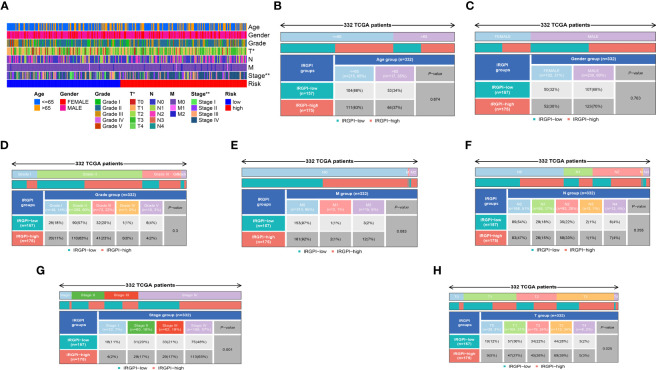
**(A)** The heatmap shows the clinical correlation analysis between IRPDGPI and clinical information; **(B)** The clinical correlation analysis between IRPDGPI and age; **(C)** The clinical correlation analysis between IRPDGPI and gender; **(D)** The clinical correlation analysis between IRPDGPI and grade; **(E)** The clinical correlation analysis between IRPDGPI and M stage; **(F)** The clinical correlation analysis between IRPDGPI and N stage; **(G)** The clinical correlation analysis between IRPDGPI and stage; **(H)** The clinical correlation analysis between IRPDGPI and T stage. The symbol “*” represents a p-value less than 0.05, while “**” indicates a higher level of statistical significance, typically representing a p-value less than 0.01.

By conducting clinical correlation analysis, we assessed the predictive significance of IRPDGPI and clinical value in determining the prognosis of patients with HNSC. Compared to the clinical data, which includes factors like age, sex, grade, T, M, and N stages, the ROC curve illustrated that IRPDGPI is a superior prognostic prediction model (**Figure 3B**). Furthermore, the DCA plot was utilized to assess the predictive significance of IRPDGPI (**Figure 5H**). To explore a better prognostic model for HNSC, an HNSC nomogram was constructed to predict survival rates (**Figure 5I**). Additionally, we investigated the association between IRPDGPI and clinical data in individuals diagnosed with HNSC. A significant correlation was observed between the stage and T stage and the IRPDGPI, with a P value of 0.05. There was no strong correlation between the IRPDGPI and factors such as age, gender, grade, M stage, and N stage. (P>0.05) (**Figures 6A–H**).

### Enhancement of functional enrichment in modules of co-expressed immune genes

**Figure 7:**
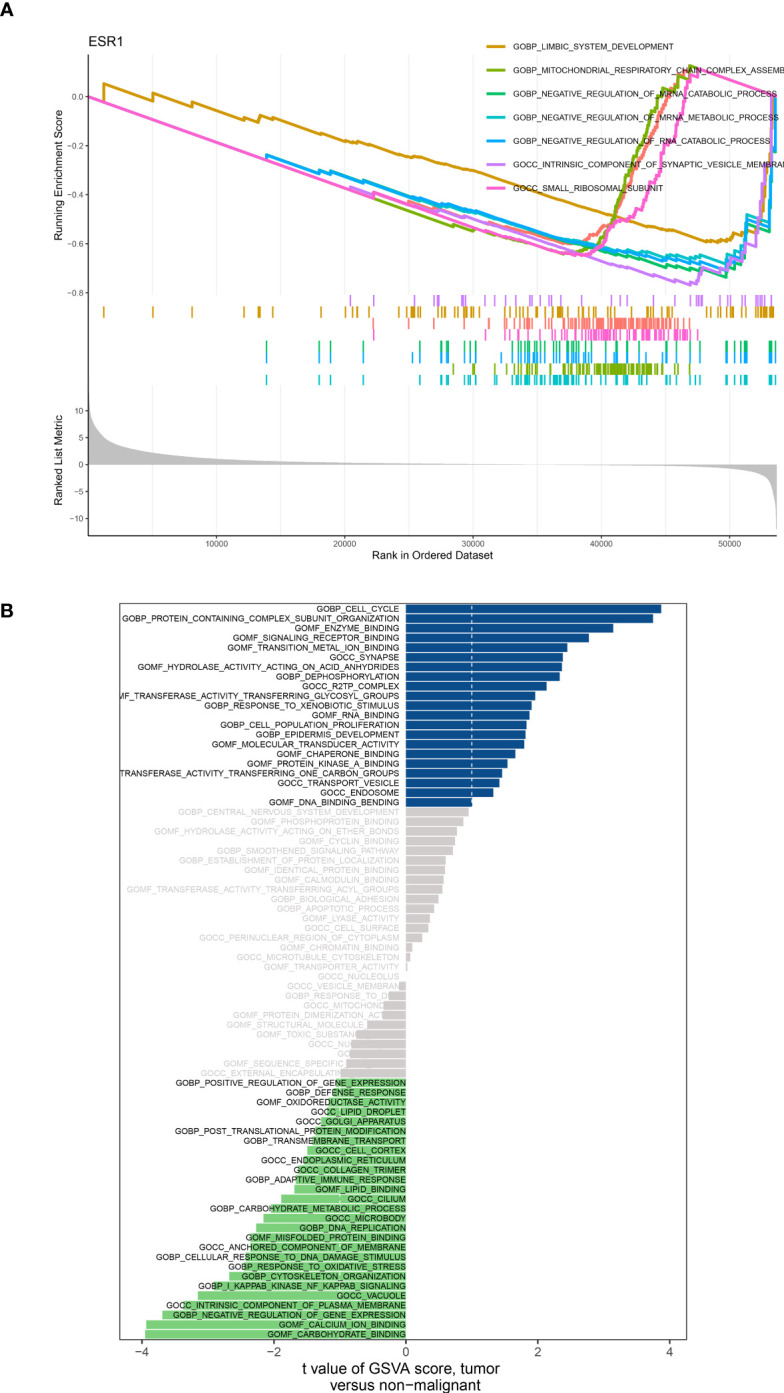
**(A)** GSEA analysis based on the different expression level of ADM; **(B)** GSVA analysis based on the different expression level of ADM.

Based on the analysis of differential expression and the predictive model for prognosis, we have identified ADM as a potential crucial gene in the HNSC cohort. Hence, we conducted an analysis of GSEA and GSVA with respect to ADM. Specifically, the GSEA enrichment analysis revealed that ADM was highly enriched in various pathways including complement activation, regulation of complement activation, blood microparticle, RNA binding involved in posttranscriptional gene silencing, immunoglobulin complex, regulation of humoral immune response, B cell-mediated immunity, humoral immune response mediated by circulating immunoglobulin, and humoral immune response. Furthermore, an examination was conducted on the particular signaling pathways associated with the ADM, investigating the potential molecular mechanisms that impact the development and advancement of lung cancer (**Figure 7A**). According to the GSVA results, the distinct expression groups of ADM exhibited significant enrichment in various signaling pathways including adaptive immune response, apoptosis, biological adhesion, carbohydrate metabolism, cell cycle, cell population growth, cellular response to DNA damage, central nervous system development, and cytoskeleton organization (**Figure 7B**).

### The evaluation of the ADM in SCC-25 and NOK-SI cell line

**Figure 8:**
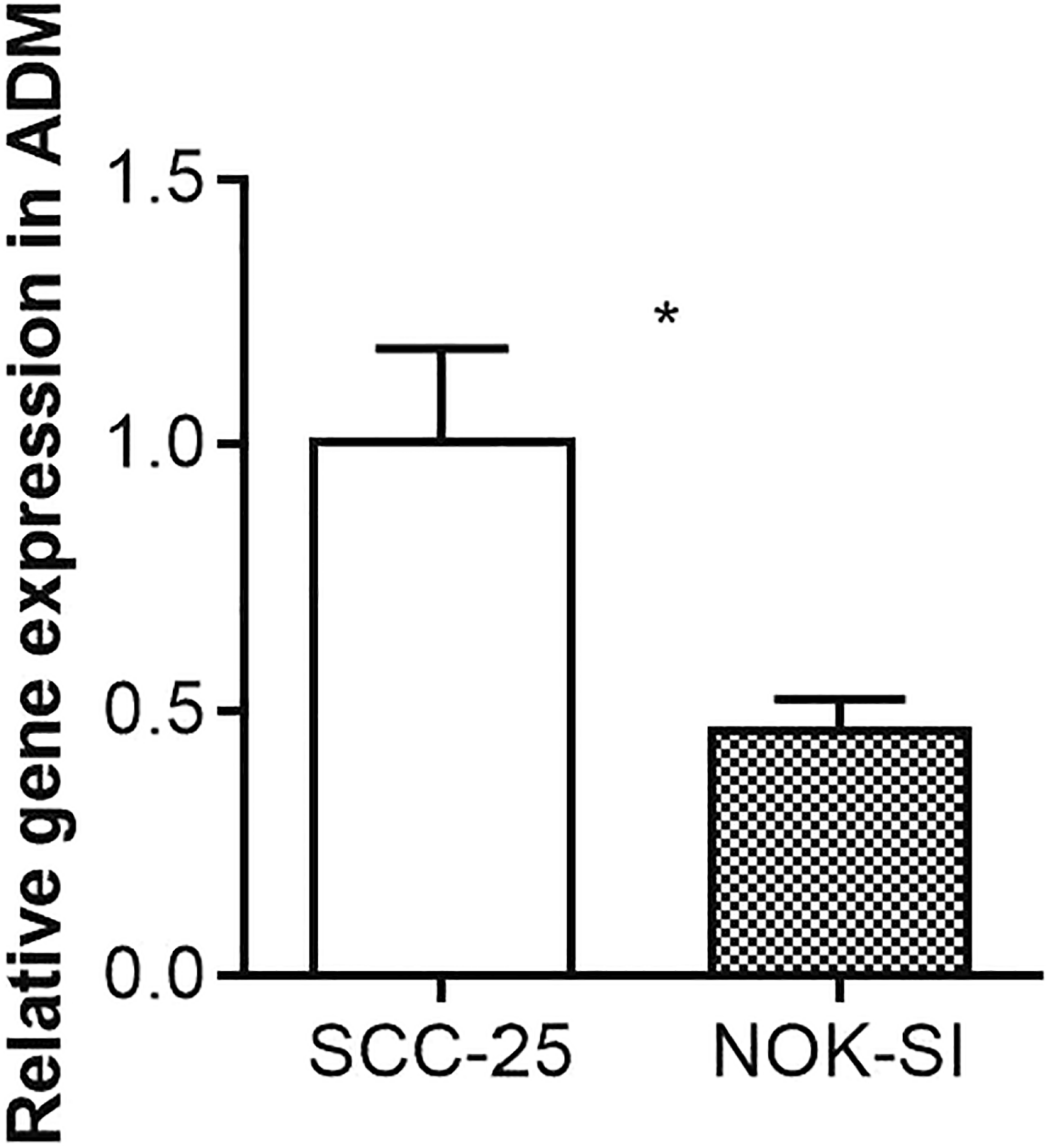
Quantitative Real-Time PCR Analysis of ADM Expression in SCC-25 and NOK-SI Cell Lines. The symbol “*” denotes statistical significance with a p-value less than 0.05.

The expression levels of Adrenomedullin (ADM) in SCC-25 and NOK-SI cell lines were quantitatively evaluated using real-time PCR. Our results showed a distinct difference in ADM expression between these two cell lines. In the SCC-25 cell line, which is derived from oral squamous cell carcinoma, the ADM expression level was significantly elevated. Relative quantification using the 2^-ΔΔCt method demonstrated an increase in ADM mRNA expression in SCC-25 cells, in comparison to the NOK-SI cell line. Conversely, the NOK-SI cell line, comprising spontaneously immortalized human oral keratinocytes derived from normal tissue, showed relatively lower levels of ADM expression (**Figure 8**).

## Discussion

In addition to similar symptoms such as swelling, bleeding, tooth movement, and deep periodontal pockets, HNSC presents clinically as advanced periodontal disease (27). Consequently, periodontitis and HNSC pose a huge health threat and cost an expensive. Activation of the immune system can enhance antitumor immune responses, providing benefits for individuals with cancer through the use of immunotherapy (28). In spite of the current enthusiasm for immunotherapy, it is unlikely that anyone immunotherapy can significantly alter HNSC (29). Although HNSC is an acknowledged condition that triggers an immune response, the virus can avoid the immune system by reducing the activity of human leukocyte antigen class I. This, in turn, induces T cell apoptosis through the production of Fas ligands and the secretion of immunosuppressive cytokines, etc (30). There is still much work to be done in the field of immunotherapy for HNSC patients. This study investigates periodontal disease-related genes and reveals that periodontal disease is closely related to HNSC. In addition, we investigated genes associated with the immune system. The findings unveiled a notable association between these genes and both periodontal disease and HNSC. There are notable variations in the expressions of various immune-related genes associated with periodontal disease between individuals with HNSC and healthy individuals. Consequently, we utilized univariate Cox regression, lasso regression, and multivariate Cox regression to establish the IRPDGPI, subsequently categorizing the subjects into two research cohorts according to the HNSC classification. The prognosis of patients with HNSC was found to be closely associated with five immune genes, namely ADM, AIMP1, STC1, and CDKN2A. Moreover, the analysis of differential expression indicated notable variations in the expression of all seven genes associated with IRPDGPI among individuals diagnosed with HNSC.

Considering that only a portion of individuals who undergo immune therapy experience positive therapeutic outcomes and prolonged survival, a guide for immunotherapy was created utilizing gene profiles related to tumor immunity in patients with HNSC. To investigate the traits of the two groups, an additional study was carried out. The relationship between IRPDGPI and prognosis in patients with HNSC is strong, suggesting that immunotype could serve as a predictive biomarker. Within the microenvironment of the tumor, various components exist including tumor cells, noncancerous cells, vascular structures, the extracellular matrix, and additional substances. The tumor microenvironment is also influenced by various types of immune cells that have a significant impact. In recent years, evidence has emerged suggesting that tumor cell characteristics can be used to evaluate immune therapy effectiveness and predict clinical outcomes (31). Various immune cell constituents exhibited notable variations among the genes associated with IRPDGPI, encompassing memory B cells, CD8+T cells, memory CD4+T cells, macrophages, and neutrophils. Natural killer cells, also known as NK cells, play a vital role in the immune system by inhibiting the proliferation of both tumor cells and virus-infected cells. As a result of bortezomib’s capabilities to increase the sensitivity of HNSC cells to NK cell-mediated killing, it may improve the effectiveness of current cancer treatments. Enrichment analysis revealed that immune-related genes associated with periodontal disease were enriched in specific pathways, including processes related to the muscular system, muscle contraction, sliding of muscle filaments, sarcomeres, myofibrils, contractile fibers, structural components of muscles, and binding of actin and alpha-actinin. Additionally, to investigate the predictive significance of IRPDGPI in HNSC individuals, we conducted DCA and ROC analyses. The findings indicated that IRPDGPI is regarded as a superior prognostic forecasting model in comparison to the clinical data, encompassing age, sex, grade, T, M, and N stage. The analysis of clinical correlation subsequently revealed a strong association between the T stage, stage of HNSC patients, and IRPDGPI. In addition, we also constructed the nomogram by integrating the clinical information and IRPDGPI to obtain a better prognostic prediction tool.

Nevertheless, the study also presents certain constraints that must be addressed in subsequent endeavors. The bioinformatics analysis without the proper assays may be not very persuasive (32). Furthermore, additional comprehensive examination is necessary to further validate the significance of the genes implicated in the prognostic forecasting model (33). Finally, the different methods may also lead to bias. It is necessary to confirm the role of risk models in the larger cohort of HNSC (34, 35).

## Conclusion

In HNSC, the development of immunotherapy could potentially focus on targeting immune-related genes such as ADM, CXCL5, FGF9, AIMP1, STC1, and CDKN2A. Furthermore, CXCL5, ADM, FGF9, AIMP1, STC1, and CDKN2A might have a significant impact on the development of HNSC caused by periodontal disease. In conclusion, we have found that ADM could potentially play a crucial role in the development of HNSC caused by periodontal disease. Thus, this research provides a theoretical basis for HNSC induced by periodontal disease immunotherapy.

## Data availability statement

The original contributions presented in the study are included in the article/supplementary material. Further inquiries can be directed to the corresponding author.

## Author contributions

YF: Software, Writing – original draft. YZ: Methodology, Writing – original draft.
